# Environmentally Friendly Synthesis of Poly(3,4-Ethylenedioxythiophene): Poly(Styrene Sulfonate)/SnO_2_ Nanocomposites

**DOI:** 10.3390/polym13152445

**Published:** 2021-07-25

**Authors:** Ana M. Díez-Pascual

**Affiliations:** Universidad de Alcalá, Facultad de Ciencias, Departamento de Química Analítica, Química Física e Ingeniería Química, Ctra. Madrid-Barcelona, Km. 33.6, 28805 Alcalá de Henares, Madrid, España (Spain); am.diez@uah.es; Tel.: +34-918-856-430

**Keywords:** PEDOT:PSS, SnO_2_ nanoparticles, green synthesis, thermoelectrical properties, mechanical properties, optical transparency

## Abstract

Conductive poly(3,4-ethylenedioxythiophene):poly(styrene sulfonate) (PEDOT:PSS) is widely used for practical applications such as energy conversion and storage devices owing to its good flexibility, processability, high electrical conductivity, and superior optical transparency, among others. However, its hygroscopic character, short durability, and poor thermoelectric performance compared to inorganic counterparts has greatly limited its high-tech applications. In this work, PEDOT:PSS/SnO_2_ nanocomposites have been prepared via a simple, low cost, environmentally friendly method without the use of organic solvents or compatibilizing agents. Their morphology, thermal, thermoelectrical, optical, and mechanical properties have been characterized. Electron microscopy analysis revealed a uniform dispersion of the SnO_2_ nanoparticles, and the Raman spectra revealed the existence of very strong SnO_2_-PEDOT:PSS interactions. The stiffness and strength of the matrix gradually increased with increasing SnO_2_ content, up to 120% and 65%, respectively. Moreover, the nanocomposites showed superior thermal stability (as far as 70 °C), improved electrical conductivity (up to 140%), and higher Seebeck coefficient (about 80% increase) than neat PEDOT:PSS. On the other hand, hardly any change in optical transparency was observed. These sustainable nanocomposites show considerably improved performance compared to commercial PEDOT:PSS, and can be highly useful for applications in energy storage, flexible electronics, thermoelectric devices, and related fields.

## 1. Introduction

**Scheme 1:**
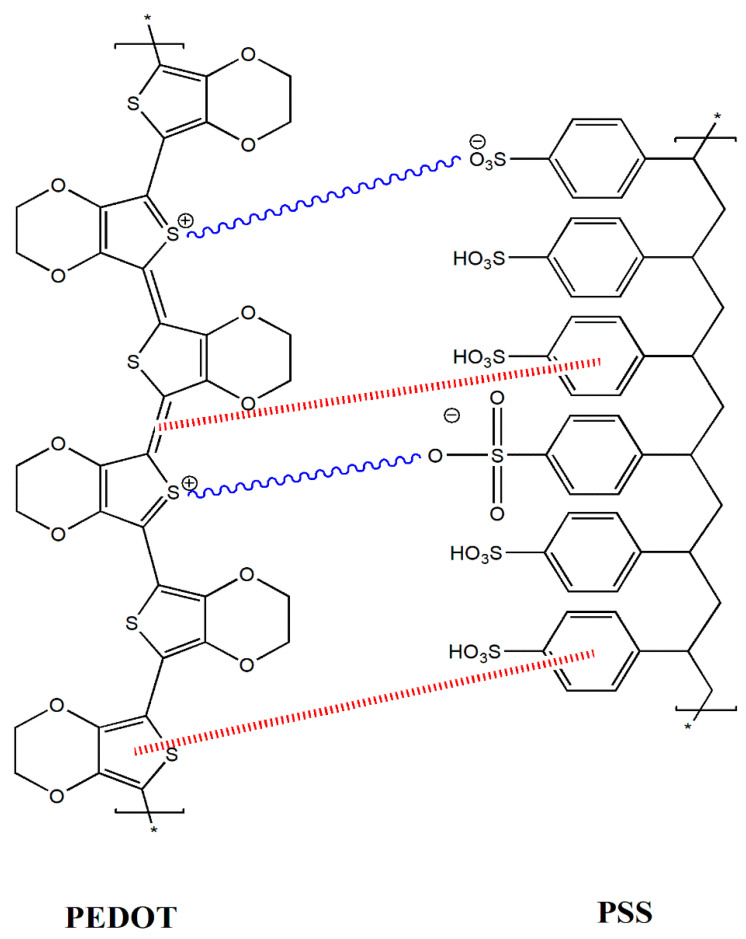
Chemical structure of PEDOT:PSS showing the electrostatic interactions between positively charged thiophene rings and sulphonate groups (blue) and π–π interactions between double bonds and thiophene rings of PEDOT and benzene rings of PSS (red).

Conducting polymers constitute a type of organic polymers that can be semiconductors or display metallic conductivity, and usually present beneficial properties for processing (solubility and good film forming ability), which makes them suitable for a comprehensive range of applications in energy storage and conversion electronic [1,2]. Poly(3,4-ethylenedioxythiophene):poly(styrene sulfonate) (PEDOT:PSS) is the most successful conducting polymer in terms of practical applications. It is a polyelectrolyte comprising negatively charged insulating PSS and positively charged electrically conducting conjugated PEDOT. The positively charged thiophene rings of PEDOT can interact with negatively charged sulfonyl groups of PSS via electrostatic interactions. Furthermore, the double bonds of PEDOT and its aromatic thiophene rings can interact with aromatic rings of PSS via π–π stacking (Scheme 1). PSS polymer anions can stabilize conjugated polymer cations in water and some polar organic solvents. Consequently, it possesses outstanding properties, such as water solubility, good processability, high electrical conductivity, superior optical transparency in the visible light range, flexibility, and good physical and chemical stability in air. However, it presents some drawbacks such as acidity, hygroscopic character, and heterogeneous electrical properties, which lead to short durability and limit device efficiency [3]. Furthermore, its thermoelectric performance is poorer than that of inorganic counterparts [4]. The methods and mechanisms for enhancing the thermoelectric properties of neat PEDOT:PSS and its potential applications have been reviewed [5,6].

In this regard, several nanocomposites made up of this conducting polymer and inorganic or carbon-based nanomaterials have been recently developed [7,8,9]. Among various approaches to prepare polymeric composites, blending, mixing, and in situ polymerization are the most commonly used. Considering that both PEDOT and PSS are semi-crystalline polymers with no observable melting temperature, it is difficult to process or modify them by a conventional melting process. Solution mixing is an appropriate strategy, though the solid content of PEDOT:PSS aqueous is low [10]. Generally, the resulting mixture is deposited on a flexible substrate or rigid surface by spraying, drop-casting, and rotary coating. For instance, Zhang and coworkers [11] prepared Bi_2_Te_3_/PEDOT:PSS nanocomposites by a two-step drop-casting method: first, ball-milled Bi_2_Te_3_ powders were dispersed in alcohol and drop casted onto a glass substrate. Then, PEDOT:PSS was dropcasted on the Bi_2_Te_3_ film. The resulting nanocomposites showed improved conductivity, up to 22 S cm^−1^ for the highest filler loading. Xiong and coworkers [12] prepared similar nanocomposites through three different means: layer-by-layer (drop-casting Bi_2_Te_3_ nanowires and PEDOT:PSS layer), hybrid (dispersing Bi_2_Te_3_ nanowires in aqueous PEDOT:PSS) and pellet structures (tableting a Bi_2_Te_3_ pellet and drop-casting PEDOT:PSS in it). The hybrid method attained the highest electrical conductivity since it led to a compact film, where PEDOT:PSS and the nanowires were very close to each other. The Seebeck coefficient for the three abovementioned methods was 14.9, 16.3 and 56.8 mV K^−1^, respectively. It was confirmed that PEDOT:PSS enhanced the carrier concentration and Bi_2_Te_3_ improved the carrier mobility. Yee et al. [13] synthesized Te nanorods in the presence of PEDOT:PSS. The nanocomposites had an electrical conductivity of 19.3 S cm^−1^ and a Seebeck coefficient of 163 mV K^−1^ while kept the matrix thermal conductivity. Yu et al. [14] mixed single-walled carbon nanotubes (SWCNTs) with PEDOT:PSS and achieved a very high electrical conductivity, close to 10^3^ S cm^−1^ at room temperature, and a Seebeck coefficient in the range of 27–41 mV K^−1^. The author suggested that the excellent thermoelectric properties could be attributed to the junction formed by SWCNTs and PEDOT:PSS, which can filter low-energy electrons. Graphene/PEDOT:PSS nanocomposites have also been prepared via inject printing, spraying or depositing onto an electrode in solar cells [15]. The electrical conductivity was as high as 1160 S cm^−1^ with only 5% of reduced graphene oxide.

A few studies also prepared PEDOT:PSS nanocomposites via in situ polymerization of the EDOT monomer. For instance, Au nanoparticles and G were mixed with this polymer via a two-stage process [16]: first, graphene was synthesized by electrolytic exfoliation of graphite in an aqueous PSS solution. Then, HAuCl_4_ and EDOT monomer were added to the former dispersion, and Au nanoparticles and PEDOT were formed via in situ reduction of HAuCl_4_ and oxidative polymerization of EDOT. Nevertheless, there are still several issues that need to be further investigated to expand the application of PEDOT:PSS-based nanocomposites, such as optimization of the nanofiller distribution, orientation, and interfacial interaction with the matrix to improve the thermoelectric properties.

On the other hand, in the last decade strong effort has been focused on the development of environmentally friendly polymers and polymer-based composites. Traditional polymers are produced from fossil fuels and display high persistence in the environment, which leads to sustainability issues related to petroleum depletion and waste management. Current regulations on the recyclability of materials and environmental requirements have impelled industries to manufacture polymer composites via alternative green methods. Furthermore, it is strongly recommended to consider this issue at the initial stages of any manufacturing process. In this regard, plasma treatment has become an interesting technology for a diversity of purposes, including surface cleaning and modifications, thin-film deposition, food safety, and so forth [17,18]. Plasma-activated processes have been demonstrated as excellent paths for the formation of thin films at both low temperature and atmospheric pressure [19]. In addition, plasma, which comprises electrons, ions, excited atoms and molecules, radicals and photons, enable the synthesis of materials difficult to be obtained via other technics. Thus, several polymers have been synthesized via in situ polymerization initiated by plasma treatment [20,21].

Tin oxide (SnO_2_), with a tetragonal rutile structure (cassiterite phase), is an n-type direct gap semiconductor (E_g_ = 3.6 eV at 300 K) that presents up to 97% transparency across the visible spectrum [22]. Thin films of SnO_2_ were reported to demonstrate high conductivities of 10^2^–10^3^ S/cm [23], as well as photocatalytic and antibacterial properties [24]. Thermal and physical deposition, hydro/solvothermal process, spray-pyrolysis, assisted self-assembly, oil-in-water microemulsion, and template-assisted synthesis are regularly employed to synthesis one-, two-, and three-dimensional SnO_2_ nanostructures. Due to their unique optical and electronic properties, these nanostructures have been widely applied in transparent conducting electrodes, solar cells, catalyst supports, gas sensors and so forth [25,26].

In this work, PEDOT:PSS/SnO_2_ nanocomposites with different SnO_2_ nanoparticle content have been developed via environmentally friendly in situ polymerization in aqueous medium using plasma-activated H_2_O_2_ as oxidant. The nanocomposites have been characterized in detail by several techniques including scanning electron microscopy (SEM), Raman spectroscopy, X-ray diffraction (XRD), electrical conductivity, Seebeck coefficient measurements, thermogravimetric analysis, UV–Vis spectrophotometry, and tensile tests. A synergetic enhancement of the thermoelectric performance, stiffness and stability of PEDOT:PSS by adding a low amount of SnO_2_ nanoparticles has been found. These sustainable nanocomposites could be of interest in the photovoltaic field of research.

## 2. Materials and Methods

### 2.1. Reagents

3,4-Ethylenedioxythiophene (EDOT) monomer (C_6_H_6_O_2_S, 97%, *M*_w_ = 142.18 g/mol, *ρ* = 1.331 g/mL), poly(sodium 4-styrenesulfonate) (PSS) ((C_8_H_7_NaO_3_S)_n_, *M*_w_ ~70,000 g/mol, *ρ* = 0.801 g/mL), hydrogen peroxide (H_2_O_2_, 35%, *M*_w_ = 34.01 g/mol, *ρ* = 1.13 g/mL), and tin (IV) oxide nanopowder (SnO_2_) (*M*_w_ = 150.71 g/mol, <100 nm particle size and specific surface area in the range of 15–25 m^2^/g) were supplied by Sigma-Aldrich (Madrid, Spain). The deionized water was produced with a Milli-Q-Water-Purification-System. All the chemicals were employed without further purification.

### 2.2. Synthesis of PEDOT:PSS/SnO_2_ Nanocomposites

First, plasma-activated H_2_O_2_ was prepared using a gliding arc plasma system as described elsewhere [27,28], comprising a plasma reactor, a high-voltage power supply, a digital oscilloscope, an optical emission spectrophotometer, and an infrared camera. A forward-vortex flow stabilized gliding arc reactor was used, which consists of a cylindrical tube with a swirl generator, placed on the opposite side of the axial gas outlet. The tangentially applied gas stream starts a vortex swirl flow along the walls of the tube, partially isolating the plasma from the walls, thus reducing its heat loss, which improves the reactor efficiency [29]. An electrical discharge was formed between two divergent electrodes connected to a high-voltage supply with high-velocity gas flow between the electrodes. Air generated by an air compressor at a flow of 10 L min^−1^ was used as the source gas. A high-voltage transformer operating at 9.3 kV was used for the generation of the discharge. A beaker containing 1 m H_2_O_2_ aqueous solution was placed on a magnetic stirrer and the nozzle of the plasma reactor was situated 5 mm from the solution surface. The plasma treatment time was 5 min.

Nanocomposites with SnO_2_ loadings of 0.5, 1.0, 2.0, 5.0 and 10 wt% were prepared by means of oxidative in situ polymerization. In a typical synthesis, 0.5 g of EDOT monomer was mixed with either 1.25 or 3.0 g of PPS (PEDOT:PSS ratios of 1:2.5 and 1:6, respectively) in 100 mL deionized water and stirred for 1 h. Subsequently, 1 mL of activated H_2_O_2_ in deionized water and the required amount of SnO_2_ were added. The dispersion was ultrasonicated for 30 min and then stirred for 24 h at 60 °C until the color of the solution changed from clear to dark blue. The product was then purified by centrifugation, washed thoroughly with deionized water, poured into a glass Petri dish and dried under vacuum for 48 h.

### 2.3. Characterization Techniques

Scanning electron microscopy (SEM) images were obtained with a SU8000 scanning electron microscope (SEM, Hitachi, Ltd., Tokyo, Japan), at a voltage of 15.0 kV and emission current of 10 mA. Prior to the observations, the nanocomposite films were cryofractured in liquid nitrogen and then coated with a ~5 nm Au:Pd overlayer to avoid charge accumulation during electron irradiation.

Room temperature Raman spectra were recorded with a Renishaw Raman microscope (Renishaw plc, Gloucestershire, UK) equipped with a He-Ne laser (632.8 nm), at a laser power of 1 mW. To reduce the signal-to-noise ratio, 20 scans were obtained for each sample. The Raman spectra were processed using the WiRE 3.3 Renishaw software.

A Bruker D8 Advance diffractometer (Bruker, Karlsruhe, Germany) was used to perform the X-ray diffraction (XRD) analysis. A Cu tube was employed as the X-ray source (Cu-Kα = 1.54 Å), with a voltage of 40 kV and an intensity of 40 mA.

Silver conductive paint was used to create top and bottom contacts for the Seebeck coefficient and electrical conductivity measurements, which were carried out on a ZEM-3M8 ULVAC System (Advanced Riko, Inc., Yokohama, Japan) under RT and a helium environment. Five specimens for each composition were tested to report an average value.

Tensile tests under RT conditions were performed with an Instron 5565 Testing Machine (Instron, Norwood, MA, USA), using a 1 kN load cell and at a crosshead speed of 10 mm/min. The results reported are the mean values for six replicates.

The thermal stability of the samples was investigated via thermogravimetric analysis (TGA) with a Q50 thermobalance (TA Instruments, Barcelona, Spain), at a heating rate of 10 °C/min, from room temperature to 700 °C. After drying for 72 h, about ~5 mg of each sample were placed into an alumina pan and measured under an inert atmosphere, with a purge gas flow rate of 60 mL/min.

The optical transmittance of the films was measured at room temperature with a UV–Vis-NIR spectrophotometry (JASCO Corporation, Tokyo, Japan, model V-650), in the wavelength range of 200–950 nm.

## 3. Results and Discussion

### 3.1. Characterization of SnO_2_ Nanoparticles

**Figure 1:**
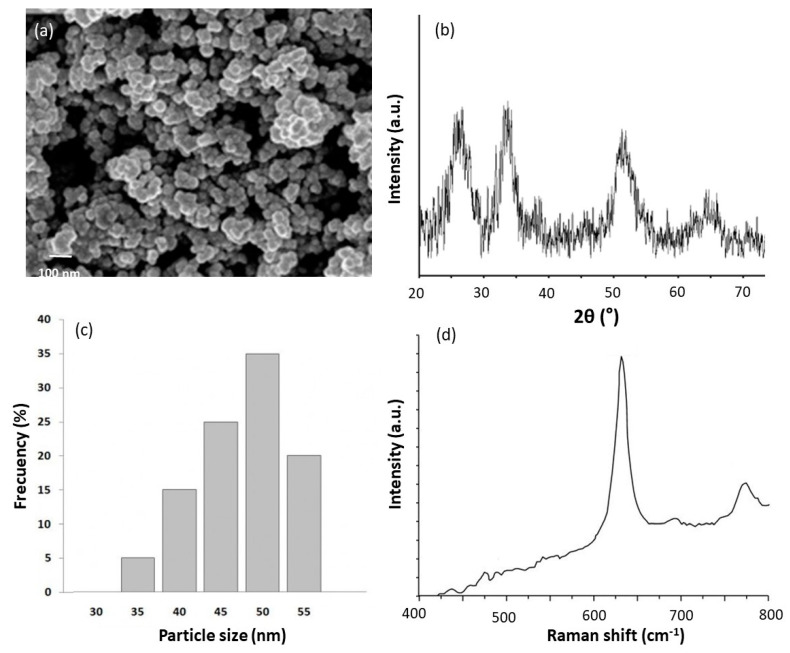
Characterization of SnO_2_ nanoparticles: (**a**) SEM image; (**b**) XRD diffractogram; (**c**) particle size distribution; (**d**) Raman spectra.

First, the pristine SnO_2_ nanoparticles were characterized by different techniques to obtain information about their structure, morphology and particle size (Figure 1). According to the SEM images (Figure 1a), the nanoparticles present quasi-spherical shape, and appear quite agglomerated, in the form of clusters comprising 2–5 particles. The surface hydroxyl groups of SnO_2_ have a strong tendency to create hydrogen bonds among nanoparticles, hence aggregation can occur, causing the formation of small clusters. The corresponding histogram shows nanoparticles with sizes ranging from 35 to 55 nm, and a mean diameter value of 47.5 nm. XRD analysis of the nanoparticles (Figure 1b) revealed four characteristic diffraction peaks at 2θ angles of 26.7°, 33.9°, 38.0°, 51.8° and 66.1°, which could be indexed to (110), (101), (200), (211), and (301) diffraction planes, respectively, hence corroborating the cassiterite crystal phase with tetragonal rutile structure (JCPDS No. 41-1445), space-group symmetry of P4_2_/mnm [30]. The unit cell consists of two Sn atoms and four O atoms. Each Sn atom is located among six O atoms which form a regular octahedron. O atoms are surrounded by three Sn atoms forming a triangle. The average crystallite size obtained from the full width at half maximum (FWHM) of the 110 plane according to the Scherrer formula [31] was 3.6 nm. On the other hand, the most important vibrations in the Raman spectra (Figure 1d) appear at 631, 691 and 767 cm^−1^, related to the A_1g_, B_1g_, and B_2g_ nondegenerated modes, respectively, in which oxygen atoms vibrate in the plane perpendicular to the c axis. The spectra confirm high SnO_2_ purity. Furthermore, according to literature [32], the positions observed correspond to nanoparticles with average crystallite sizes in the order of 3.5–4.5 nm, in good agreement with the results from XRD analysis.

### 3.2. Morphology of the Nanocomposites

**Figure 2:**
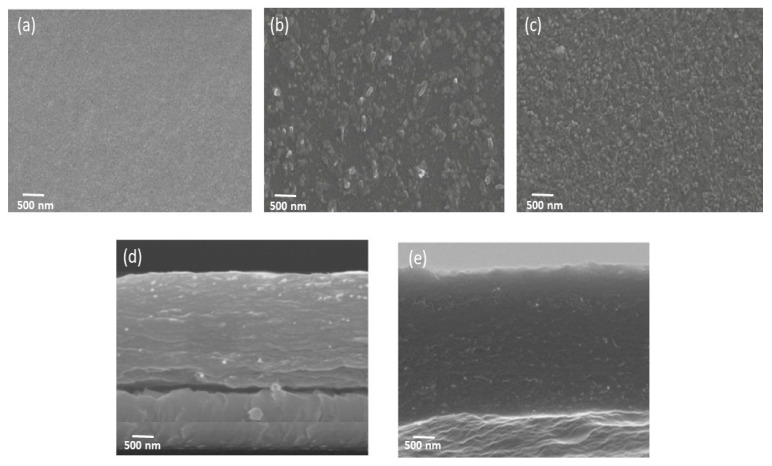
SEM images of neat PEDOT:PSS (**a**), PEDOT:PSS/SnO_2_ nanocomposites with 5.0 wt% SnO_2_ (**b**,**d**) and 10.0 wt% SnO_2_ (**c**,**e**), for a PEDOT:PSS ratio of 1:6. (**a**–**c**) are micrographs of the upper surfaces and (**d**,**e**) correspond to the cross section.

The surface morphology of the nanocomposites was examined by SEM, and representative images of PEDOT:PSS (1:6) and nanocomposites with 5.0 and 10.0 wt% SnO_2_ (both surface and cross section micrographs) are shown in Figure 2. Similar images were found for the nanocomposites based on PEDOT:PSS with ratio of 1:2.5. The raw polymer shows a uniform and smooth surface, consistent with a low level of crystallinity, as previously reported [33]. Random and homogeneously dispersed nanoparticles can be observed in the cross section and at the surface of the nanocomposites, without aggregates. This indicates that the in situ oxidative polymerization in aqueous medium carried out in this work was beneficial for disrupting the aggregation of the SnO_2_, thus leading to a uniform dispersion of the nanoparticles without the need for surface functionalization treatments or compatilibizing agents, making the fabrication process of these nanocomposites facile, inexpensive, and environmentally friendly. Upon increasing SnO_2_ concentration, the surface becomes rougher. This increased surface roughness could be indicative of strong SnO_2_-PEDOT:PPS interactions, as reported previously for other conductive polymers filled with nanoscale fillers [34]. The more intense the interactions, the coarser the surface. In fact, the H-bonding interactions between negatively charged sulphonate groups of PSS and surface OH groups of SnO_2_ could promote the formation of a distorted polymeric layer on the nanoparticle surface, and this could be an advantage for certain engineering applications, since rough surfaces generally wear more rapidly and have greater friction coefficients than smooth surfaces. Moreover, roughness may favor adhesion. On the other hand, no voids or discontinuities are found between the nanoparticles and the polymer, hinting towards good compatibility between the two phases.

### 3.3. Raman Spectra of the Nanocomposites

**Figure 3:**
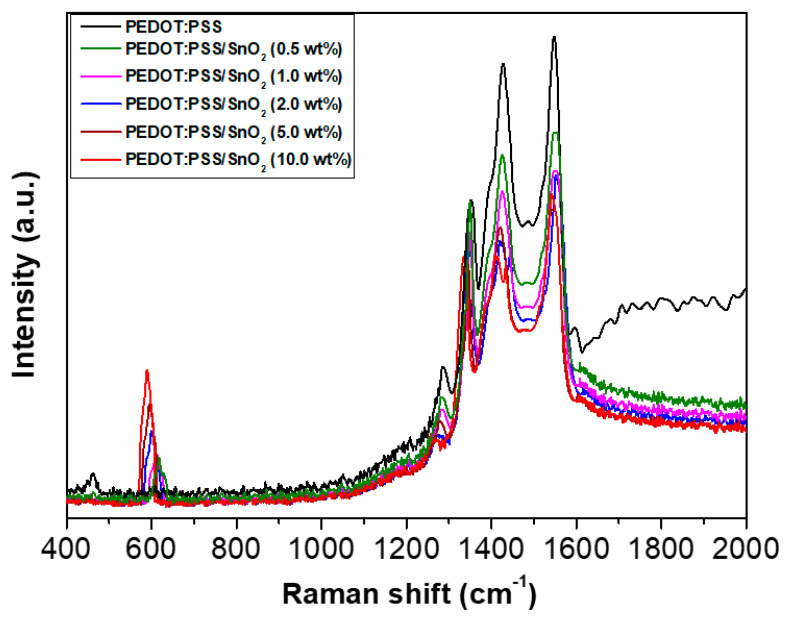
Raman spectra of neat PEDOT:PSS (1:6) and the nanocomposites with different SnO_2_ content.

To acquire further insight about the interactions between PEDOT:PSS and the nanoparticles, the nanocomposites were characterized by Raman spectroscopy, and typical spectra are shown in Figure 3. The spectrum of the neat polymer shows four characteristic stretching vibrations [35]: The C–C inter-ring stretching at 1290 cm^−1^, the single C–C stretching at 1360 cm^−1^, the C=C symmetrical stretching at 1430 cm^−1^, and the C=C antisymmetric stretching at 1560 cm^−1^. Regarding the nanocomposites, a reduction in the intensity of the PEDOT:PSS bands can be clearly observed, combined with a shift in their position. Moreover, a new peak appears in the range of 603–580 cm^−1^, related to the A_1g_ vibrational mode of the nanoparticles, as shown in Figure 1d. This peak shows increased intensity and shifts gradually towards lower wavenumber with incresing SnO_2_ loading. Thus, for the nanocomposite with 10 wt% SnO, the peak maximum is shifted by about 38 cm^−1^ compared to the raw nanoparticles. All these phenomena are ascribed to the adsorption of the polymeric chains onto the nanoparticle surface via H-bonding interactions [33]. The clear shift of the Raman bands with increasing SnO_2_ loading is indicative of stronger interactions between the nanocomposite components. Similar behavior of shift of the Raman bands has been reported for PEDOT:PSS mixed with graphene and multi-walled carbon nanotubes via in situ polymerization [36]. Moreover, some authors proposed conformational changes in the polymer coils due to interactions with embedded nanoparticles [37]. The Raman shift indicates a weakness of the C=C stretching bond, hinting that the conformation of PEDOT changes from a benzoid structure (coil conformation) to a quinoid structure (linear conformation). These changes can affect the vibrational bands of specific functional groups, which leads to variations in the Raman signal. Furthermore, it was found that the higher the PSS ratio, the more pronounced the Raman shift, likely due to the increased H-bonding interactions, since more negatively charged sulfonate groups would be prone to interact with the SnO_2_.

### 3.4. Thermoelectric Performance of PEDOT:PSS/SnO_2_ Nanocomposites

**Figure 4:**
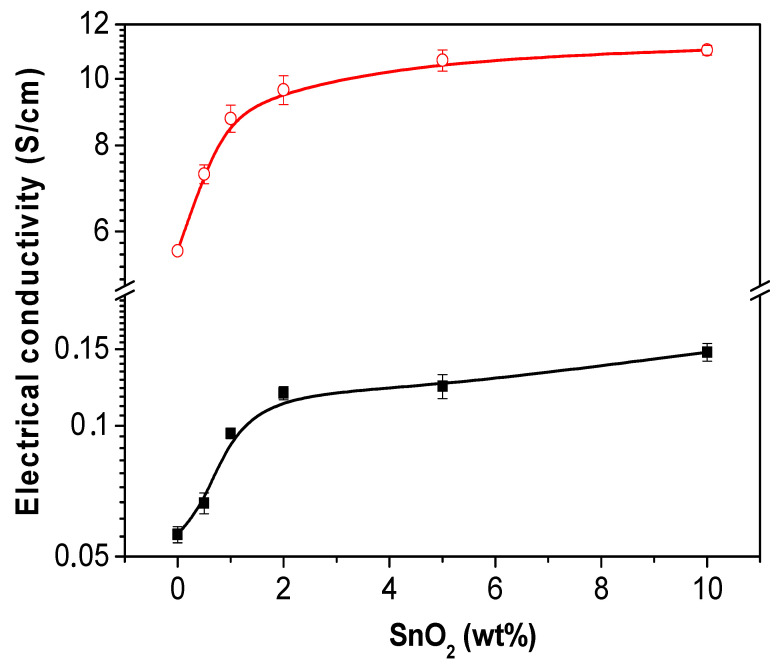
Electrical conductivity of PEDOT:PSS/SnO_2_ nanocomposites vs. SnO_2_ loading for PEDOT:PSS ratios of 1:6 (black solid squares) and 1:2.5 (red open circles).

Figure 4 shows the effect of SnO_2_ concentration and PEDOT:PSS weight ratio on the electrical conductivity of the nanocomposites. Remarkably, the addition of small quantities of SnO_2_ nanoparticles causes a significant increase in the electrical conductivity of the polymer matrix, by up to 3-fold enhancement for a PEDOT:PSS ratio of 1:6, despite the SnO_2_ nanoparticles showed a lower electrical conductivity, around 1.4 × 10^−3^ S/cm. Actually, room temperature conductivity values in the range of 5 × 10^−4^ up to 3 × 10^−3^ S/cm have been reported for SnO nanoparticles with size between 20 and 50 nm [38]. The observed improvement could be related to the strong interactions between SnO_2_ and PEDOT:PSS via H-bonding, as demonstrated from the Raman spectra. Moreover, the addition of nanoparticles can modify the Coulombic interaction between positively charged PEDOT and negatively charged PSS, thus resulting in better electrical transfer. Analogous behavior of electrical conductivity improvement has also been reported upon addition of SnO_2_ nanoparticles to other conductive polymers such as polyaniline (PANI) [39]. The conductivity in the PEDOT:PSS network depends on the proximity between polymeric chains and on the sample morphology. The morphology of PEDOT:PSS has a particular core-shell grain structure composed of conductive and tangled PEDOT-rich cores surrounded by shells with excess PSS chains [40]. PEDOT has very short and lightweight segments compared with those of PSS, which acts as a polymer matrix. PSS acts as an obstacle to the conduction of the carriers. The conduction takes place within the grains with hops from one PEDOT segment to another according to the hopping model [41]. Therefore, it could be envisaged that the presence of the SnO_2_ has a noticeable effect on the charge hopping conduction mechanism of the conductive matrix via doping and screening effects [42]. The positively charged thiophene rings of PEDOT and the negatively charged sulphonate moieties of PSS interact via electrostatic forces (Scheme 1). These Columbic interactions could be screened through H-bond formation between the surface OH groups of the SnO_2_ and the sulphonate groups of PSS, resulting in a more compact packaging of the PEDOT chains in the nanocomposite [43], which is reflected in higher conductivity. Furthermore, the presence of SnO_2_ could induce conformational changes of the polymeric chains, in particular the transformation of the PEDOT chains from a benzoid to a quinoid structure, as inferred from the Raman spectra, and therefore become more planar, which will allow a denser packaging, hence reflected in higher conductivity [44].

On the other hand, it is found that the conductivity increases up to 2.0 wt% SnO_2_ content and then increases only marginally and tends to level off, suggesting that a percolation level has been attained at that concentration, hence further increase in the nanoparticle concentration hardly influences the conductivity. Similar behavior of percolation threshold at very low nanoparticle loading (i.e., 1–2 wt%) has been reported for poly(methyl methacrylate) (PMMA) filled with SnO_2_ doped with antimony [45], and it was claimed that the percolation threshold is affected by the size ratio between the matrix and the filler.

Regarding the effect of the PEDOT:PSS ratio, it is found that nanocomposites with a ratio of 1:6 systematically display lower conductivity values, on average about 100 times lower, which is reasonable considering that PEDOT is a conductive polymer while PPS is insulating [42]. This is also in agreement with previous studies that investigated the effect of PSS content on the electrical conductivity and found that it could be interpreted as a percolation between sites of highly conducting PEDOT:PSS complexes with a conductivity of 2.3 S/cm in a matrix of excess PSS with a low conductivity of 10^−3^ S/cm [46]. Nonetheless, the conductivity improvement is stronger for the nanocomposites with a PEDOT:PSS ratio of 1:6 compared to those with a ratio of 1:2.5 (3-fold vs. 2.3-fold enhancement, respectively). This could be explained considering that the higher the PSS content, the higher the number of H-bonds with the nanoparticles, hence the abovementioned screening effect will be stronger and the packaging of the PEDOT chains will be denser. The electrical conductivity values obtained herein are comparable to those previous reported for PEDOT:PSS nanocomposites with GO or its derivatives [7], corroborating that the approach using herein is also valuable for enhancing the matrix conductivity.

**Figure 5:**
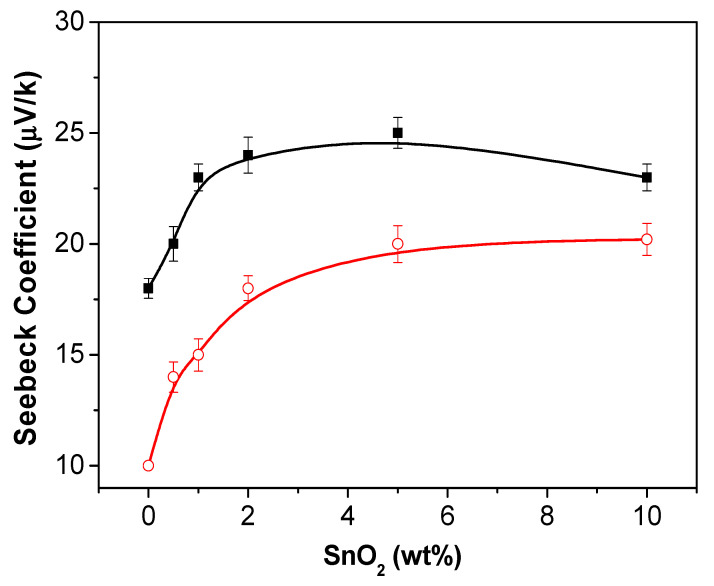
Seebeck coefficient of PEDOT:PSS/SnO_2_ nanocomposites vs. SnO_2_ loading. Symbols as indicated in Figure 4.

The thermoelectric effect is a phenomenon by which a temperature difference is directly converted to electric voltage and vice versa. The term thermoelectric effect or thermoelectricity encompasses three phenomena, known as the Seebeck effect, the Peltier effect, and the Thomson effect [47]. The Seebeck coefficient measures the magnitude of an induced thermoelectric voltage in response to a temperature difference across that material. The Seebeck coefficient data for PEDOT:PSS nanocomposites reinforced with SnO_2_ are compared in Figure 5. As can be observed, all the nanocomposites with PEDOT:PSS ratios of 1:6 and 1:2.5 have Seebeck coefficients higher than raw PEDOT:PSS. This coefficient increases sharply at low SnO_2_ loadings and then remains merely unchanged or even decreases slightly. Thus, the excessive addition of inorganic nanoparticles was ineffective for improving the thermoelectric performance. Given that the thermopower of a material depends greatly on impurities, imperfections, and structural changes, the behavior observed points towards a change in the structure of the polymeric network. Previous studies dealing with PEDOT:PSS nanocomposites reported that inorganic nanofillers can improve the Seebeck coefficient by the filtration of low-energy carriers and transporting high-energy carriers, known as “energy-filtering effect” [13,48]. Such mechanism could apply to the nanocomposites developed in this work. Thus, the strong interactions between SnO_2_ and PSS via H-bonding could result in polymeric segments orderly aligned, which improves the electrical conductivity. Moreover, the formation of a more densely packed PEDOT chains as well as a shorter π–π stacking distance would be reflected in increased Seebeck coefficient [47].

The Seebeck coefficients of nanocomposites with a PEDOT:PSS ratio of 1:2.5 are systematically lower than those with a ratio of 1:6. However, it has been previously found that the charge carrier density is independent of the PSS content [46]. Thus, it could be explained considering that the higher the PSS content, the higher the number of H-bonds between the SnO_2_ and the sulphonate groups of PSS, hence the stronger the interactions at the molecular level. This is consistent with previous studies, which found that some organic nanofillers such as graphene derivatives not only improve the electrical conductivity of conductive polymers, but also increase the Seebeck coefficient owing to the energy-filtering and ordered chains at the interphases within the nanocomposites [49]. It is important to note that the Seebeck coefficient of bulk SnO_2_, a n-type semiconductor with wide band gap, is higher than that of PEDOT:PSS, hence another reason for the results obtained. Thus, the values of Seebeck coefficient obtained herein are higher than those obtained upon addition of raw graphene [50], or small amounts of other metal oxides such as ZnO [51].

### 3.5. Thermal Stability of PEDOT:PSS/SnO_2_

**Figure 6:**
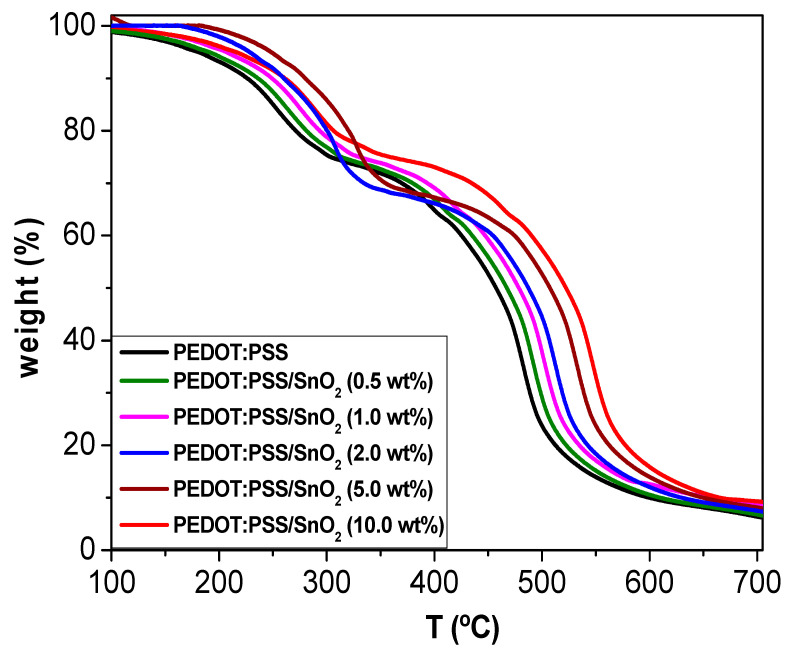
TGA curves under inert atmosphere of neat PEDOT:PSS (1:6) and the nanocomposites with different SnO_2_ loading.

The thermal stability of polymer composites is of countless interest for certain applications such as thermoelectric devices. To acquire insight about the stability of the nanocomposites upon increasing temperature, TGA measurements were accomplished under nitrogen atmosphere, and the results are shown in Figure 6 and Table 1. It has been reported that PEDOT:PSS films show a complex degradation mechanism that includes a morphological degradation in the range of 25–130 °C, in which the ionic bonds between the PEDOT oligomers and the PSS chains start to break, and a chemical decomposition at higher temperatures [52]. As can be observed, neat PEDOT:PSS shows two breakage stages; the first weight loss between 130 and 300 °C can be attributed to the decomposition of PSS via removal of the sulphonate groups, and the second between 350 and 550 °C is ascribed to the splintering of the PEDOT and PSS backbone chains [53].

The nanocomposites also exhibit a two-step degradation process, similar to that of pristine PEDOT:PSS. With increasing SnO_2_ content, the TGA curves move gradually towards higher temperatures, and the initial degradation temperature taken at 2% weight loss (*T*_onset_) as well as the temperature of 10% weight loss (*T*_10_) and the temperature of maximum rate of weight loss (*T*_peak_) rise, together with the weight residue (*R*), corroborating higher thermal stability, and flame retardancy for the nanocomposites. The maximum improvements in these temperatures are found at 5.0 wt% SnO_2_ content. In particular, for the nanocomposite with a PEDTO:PSS ratio of 1:6, the increments in the aforementioned temperatures are 71°, 50° and 51°, respectively (Table 1). Thus, the nanoparticles regularly dispersed inside the conductive matrix can act as a barrier and delay the flow of the degradation products from the bulk of the sample to the gas phase via formation of a tortuous pathway. Additionally, they could also act as thermal shielding materials to isolate the PEDOT:PSS chains from the heat. Similar behavior has been reported for other PEDOT:PSS composites reinforced with nanoclays [54], ascribed to the barrier effect of the inorganic nanofillers. Furthermore, the strong PEDOT:PSS-SnO_2_ interactions could restrain the rotational movement of the polymeric chains, thus decreasing molecular movement, which is reflected in better thermal stability. This could explain the fact that the stability is systematically higher for the nanocomposites with higher PSS content (Table 1), which have more H-bonds, hence stronger filler–matrix interactions, which would result in a more effective barrier effect. However, for both PSS contents, the nanocomposites with 10 wt% SnO_2_ display slightly lower stability than those with 5.0 wt% content, signifying that the barrier effect imposed by the nanoparticles has leveled off. Improved thermal stability upon addition of SnO_2_ nanoparticles has also been reported for other polymeric matrices such as polyvinyl alcohol (PVA) [55] or polyurethane (PU) [56], also ascribed to the strong intermolecular interactions between the components.

### 3.6. Tensile Properties of PEDOT:PSS/SnO_2_ Nanocomposites

**Figure 7:**
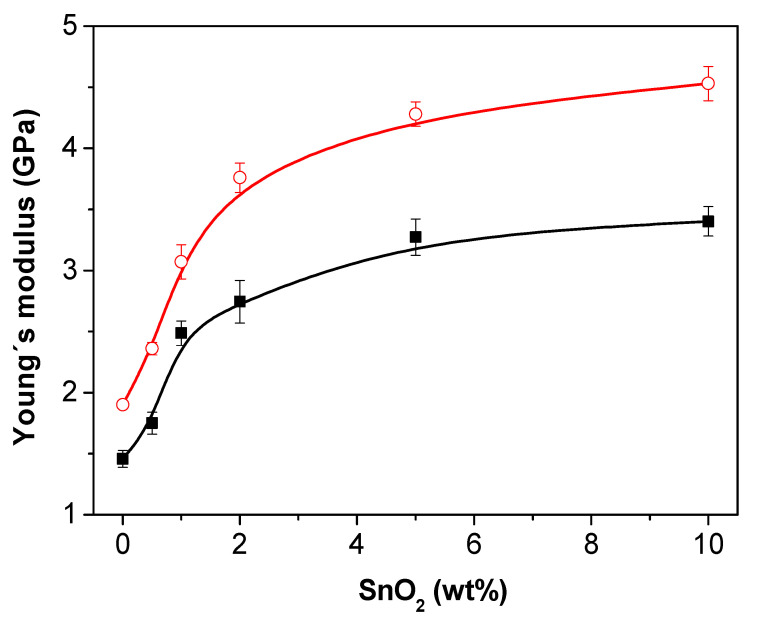
Young´s modulus of PEDOT:PSS/SnO_2_ as a function of nanoparticle loading. Symbols as indicated in Figure 4.

To obtain information about the mechanical properties of the nanocomposites, tensile tests were performed, and the Young’s modulus obtained from the stress-strain curves are shown in Figure 7. Pristine PEDOT:PSS (1:6) has a Young’s modulus of about 1.5 GPa, while that with a ratio of 1:2.5 is slightly higher, close to 2 GPa, in agreement with the highest modulus of PEDOT (2.6 GPa [57]) compared to PSS. In both cases, the addition of SnO_2_ causes an increase in stiffness, by more than two-fold at 10 wt% loading for that with a ratio of 1:2.5, the reinforcement effect being more pronounced at low loadings. The strong modulus increase found, especially at low SnO_2_ contents, demonstrates the great reinforcing efficiency of the inorganic nanoparticles, likely due to the combination of a random and homogenous dispersion within the matrix and a very strong interfacial adhesion due to numerous hydrogen bonds, as mentioned above, together with the high modulus of these nanoparticles (about 150 GPa [58]).

Indeed, it has been demonstrated that the mechanical properties of nanoparticle-polymer nanocomposites are governed by interactions on the nanoscale between the nanoparticles and the matrix [59]. Furthermore, other effects such as morphology, nucleation efficiency, molecular confinement and interfacial area and interaction/adhesion at the interphase can also be decisive in improving the stiffness of the nanocomposites. The interphase properties depend on the nanoparticle size, density, and specific surface area. Given that the nanoparticles employed display high specific surface area and very small size (Figure 1), they lead to a large interphase with the matrix and hence have very strong stiffening effect.

Taking into account the reported Young’s modulus for SnO_2_ [58], the theoretical Young´s modulus values for the nanocomposites were estimated by the modified rule of mixtures [60]: *E*_c_ = (*η E_f_* − *E_m_*) *V_f_* + *E_m_*, where *η* is a particle-strengthening factor (0.2 for randomly oriented fillers, *E_f_* the filler modulus, *E_m_* the matrix modulus, and *V_f_* the filler volume fraction). Surprisingly, experimental data of composites with 0.5, 1.0, and 2.0 wt% are about 6, 22, and 15% higher than the calculations, that with 5.0 wt% is in perfect agreement with the predictions, while that with 10 wt% is about 35% lower than the expectations. The discrepancies are likely due to the fact that the model assumes perfect adhesion between the phases and that stress is transferred via a shear mechanism. However, fatigue, stress amplification, strain localization, and other phenomena can take place at the interphase, which are not considered in the equations [61], and this could account for the differences between the theory and experimental data. Furthermore, the model assumes that the modulus of each phase is independent and remains unchanged by the presence of the other components, while at higher loadings, interactions among nanoparticles can take place, which reduce the load transfer efficiency.

The differences between the behavior found at low and higher SnO_2_ nanoparticle loading can be rationalized as follows: The nanoparticles restrict the mobility and deformation of the matrix by introducing a mechanical constraint. The restriction happens because of an effective attraction potential between chain segments and the repulsive potential that the polymer is subjected to when it is close to solid nanoparticles. This restriction in mobility should be limited to a very small volume (for a low volume fraction) since the thickness of the interphase is usually about 1 nm or smaller and the content of particles added in composites is usually small. However, for a higher volume fraction, the elastic modulus of the confined polymer near the interfaces is much lower than that of the nanoparticles, though higher than the unconfined polymer. The trend observed herein has been previously reported for other polymers reinforced with inorganic spherical nanoparticles such as SiO_2_ [62] or WS_2_ [63]. It is noteworthy that the reinforcing effect obtained upon addition of SnO_2_ is comparable to that observed for PEDOT:PSS nanocomposites filled with carbon nanomaterials such as single-walled CNTs [64] or graphene derivatives [33], despite the higher modulus of the CNTs or graphene (about 1 TPa) compared to SnO_2_. All these facts corroborate the effectiveness of the environmentally friendly in situ polymerization process developed in this work.

**Figure 8:**
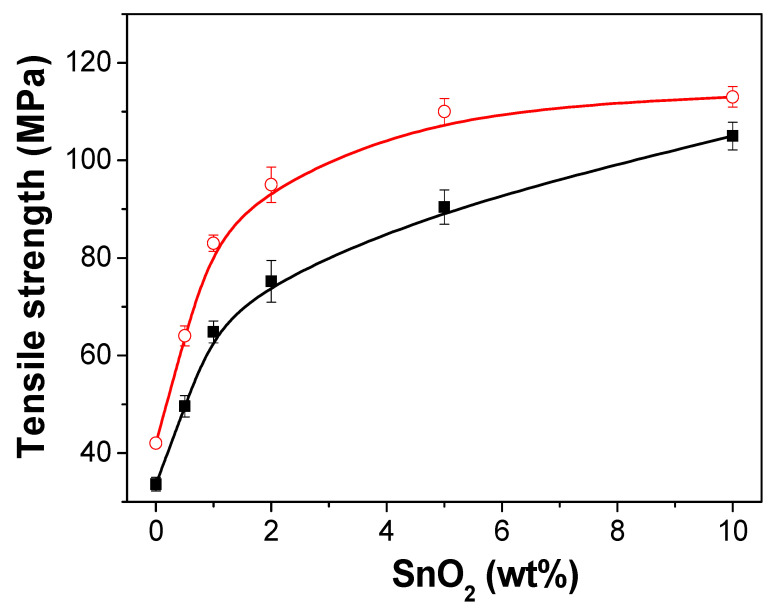
Tensile strength of PEDOT:PSS/SnO_2_ as a function of nanoparticle loading. Symbols as indicated in Figure 4.

Regarding the tensile strength (Figure 8), the trend observed is fairly similar to mentioned above for the modulus: the polymer with lower PSS content has lower tensile strength. In both cases, a very strong rise (up to 65%) in strength is found at low concentrations, and a smaller increment or even a level off at higher loadings. This increase in the tensile strength is also attributed to the strong interfacial adhesion owing to the numerous H-bonding interactions between the polymer and the nanoparticles. The strength of particulate-polymer composites also relies on the particle size, interface adhesion, and particle loading. Hard particles can affect the strength in two ways [60]. One the one hand, they can induce a weakening effect due to the stress concentration they cause. On the other hand, they have a reinforcing effect since they act as barriers to crack growth. At low loadings, the former effect typically predominates, therefore leading to a strength improvement, as found in this work.

### 3.7. Optical Properties of PEDOT:PSS/SnO_2_ Nanocomposites

**Figure 9:**
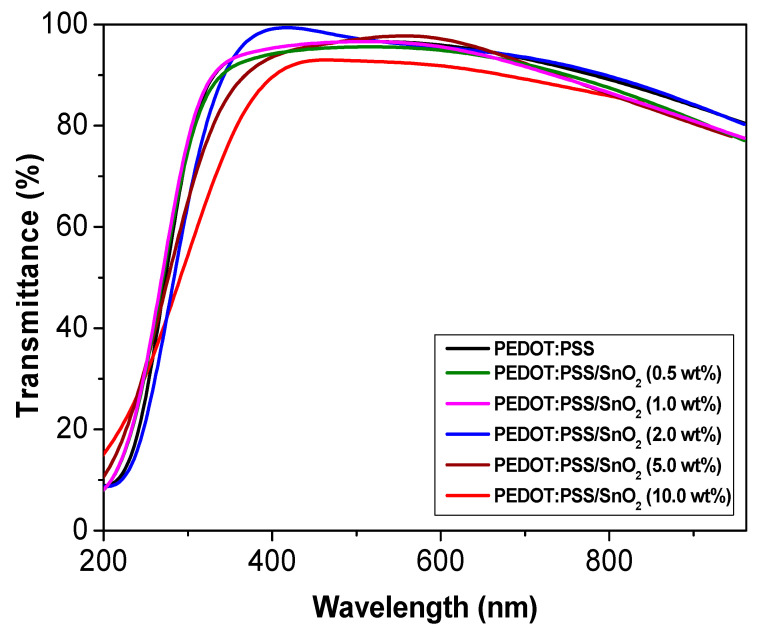
UV spectra of PEDOT:PSS (1:6) and PEDOT:PSS/SnO_2_ samples with different nanoparticle content.

UV-VIS-NIR spectra were recorded for all the samples, and the transmittance of neat PEDOT:PSS, and the nanocomposites with a PEDOT:PSS ratio of 1:6 are shown in Figure 9. Similar behavior was found for the nanocomposites with a ratio of 1:25, albeit with slightly lower values (about 3%). The neat polymer shows a maximum transmittance close to 93% at 400 nm, which decreases slightly with increasing wavelength, being about 82% at 900 nm. The lower transmittance in the NIR range has been ascribed to the bipolaron subgap transition (BST) and the free carrier effect of the PEDOT [65]. The transmittance of the nanocomposites follows similar trend, with a small a reduction in optical transmittance compared to PEDOT:PSS, by on average 4% for the nanocomposite with the highest loading. However, taking into account the instrumental resolution, the differences shown can be considered within the experimental error. Moreover, light scattering effects produced by the presence of heterogeneities at the surface can also be the origin of the observed small differences in the transmittance of the films. The nanocomposites developed in this work show higher transmittance than those incorporating carbon-based nanomaterials such as CNTs [66] or graphene [67], probably due to the very homogenous nanoparticle dispersion and the strong PSS-SnO_2_ interfacial adhesion attained via H-bonding, combined with the better transparency of SnO_2_ [22]. Furthermore, a red shift is found with increasing SnO_2_ concentration; this shift in the BST indicates an increase in the carrier concentration of the PEDOT. The BST is the photo-excited electron transition from the Fermi level to the energy level within the electrical band gap. Therefore, the red-shifted BST suggests a conformational change of the PEDOT chains from a benzoid structure to a quinoid structure, as inferred from the Raman spectra. More importantly, the values obtained for nanocomposites with SnO_2_ loadings up to 2wt% are comparable to those reported for indium tin oxide (ITO) thin films, a transparent conductive electrode widely used in solar cells, coated on a glass substrate (90%) or on flexible plastic substrates (85%) [68]. Overall, it seems that the SnO_2_ weight percentage and the PSS content can be optimized to maximize the optical transmittance.

## 4. Conclusions

In this work, nanocomposites made of conductive PEDOT:PSS, with ratios of 1:6 and 1:2.5, filled with different amounts of SnO_2_ nanoparticles have been synthesized via a facile, inexpensive, and environmentally friendly method without the need for organic solvents or compatibilizing agents. Their morphology, thermal, thermoelectrical, optical, and mechanical properties have been investigated in detail. SEM analysis revealed a homogenous dispersion of the SnO_2_ nanoparticles for all the formulations tested. Extraordinary improvements in electrical conductivity (more than 3-fold), Seebeck coefficient (about 80%), thermal stability (up to 70 and 50 °C in the onset and the maximum degradation rate temperatures, respectively), Young’s modulus (more than 2-fold) and tensile strength (about 65%) were found. These are ascribed to the strong interactions between the surface hydroxyl groups of the nanoparticles and the sulphonate groups of PSS via H-bonding. Moreover, the presence of SnO_2_ could induce conformational changes of the PEDOT chains from a benzoid to a quinoid structure, as inferred from the Raman spectra, and therefore become more planar, allowing a denser packaging, which should account for the improved performance found with increasing SnO_2_ loading. Moreover, according to the UV-VIS-NIR spectra, hardly any change in optical transparency was observed upon addition of the nanoparticles. These sustainable nanocomposites show considerably improved performance compared to commercial PEDOT:PSS, and are very promising candidates for applications in energy storage, thermoelectric devices, and high thermal applications.

## Figures and Tables

**Table 1:** Data obtained from TGA thermograms of PEDOT:PSS/SnO_2_ nanocomposites.

Sample	*T*_onset_ (°C)	*T*_10_ (°C)	*T*_peak(I,II)_ (°C)	*R* (wt%)
PEDOT:PSS	130	227	249, 479	6.8
PEDOT:PSS (1:2.5)/SnO_2_ (0.5 wt%)	136	225	249, 462	7.1
PEDOT:PSS(1:2.5)/SnO_2_ (1.0 wt%)	147	230	257, 485	7.9
PEDOT:PSS(1:2.5)/SnO_2_ (2.0 wt%)	169	246	270, 527	8.4
PEDOT:PSS(1:2.5)/SnO_2_ (5.0 wt%)	178	252	287, 545	9.7
PEDOT:PSS(1:2.5)/SnO_2_ (10 wt%)	183	258	282, 539	10.3
PEDOT:PSS (1:6)/SnO_2_ (0.5 wt%)	149	247	265, 497	7.0
PEDOT:PSS (1:6)/SnO_2_ (1.0 wt%)	174	259	279, 498	7.5
PEDOT:PSS (1:6)/SnO_2_ (2.0 wt%)	205	273	296, 534	8.0
PEDOT:PSS (1:6)/SnO_2_ (5.0 wt%)	201	277	300, 561	8.5
PEDOT:PSS (1:6)/SnO_2_ (10 wt%)	190	274	299, 573	9.7

*T*_onset_: initial degradation temperature at 2% weight loss; *T*_10_: temperature of 10% of weight loss. *T*_peak_: temperature of maximum rate of weight loss. The subscripts I and II refer to the first and second degradation stages, respectively. *R*: residue at 700 °C.

## Data Availability

The data presented in this study are available on request from the corresponding author.
